# The landscape ecological view of vertebrate species richness in urban areas across biogeographic realms

**DOI:** 10.1038/s41598-023-43896-z

**Published:** 2023-10-03

**Authors:** Chun-Wei Huang, Jia Qing Ooi, Si Ying Yau

**Affiliations:** 1 General Education Center, Ming Chi University of Technology, New Taipei City, 243303 Taiwan; 2 Department of Geography, National Taiwan University, Taipei, 10617 Taiwan

**Keywords:** Ecology, Biogeography, Ecological modelling, Urban ecology

## Abstract

Understanding how the spatial arrangement of remnant green spaces in cities complements biodiversity provides an opportunity for synergy between urban development and biological conservation. However, the geography of urbanization is shifting from Europe and North America to Asia and Africa, and more research is needed for fast-growing regions. To understand how shifting urbanization shapes biodiversity patterns, we analyzed the contribution of landscape factors in explaining vertebrate species richness in urban areas across biogeographic realms. We used variation partitioning to quantify and compare the relative importance of landscape factors (composition and configuration) and environmental factors (climate, elevation, and latitude) in explaining vertebrate species richness in landscapes with at least a million inhabitants across biogeographic realms. Our results pointed out that in the Indo-Malayan, the Afrotropical, and the Neotropical realm (on average of 16.46%) and China and India (11.88%), the influence of landscape factors on vertebrate species richness are significantly higher than that of the Palearctic and Nearctic realms (6.48%). Our findings outline the importance of landscape composition and configuration in shaping biodiversity patterns in regions with fast urban growth during the next two decades, such as Africa, Latin America, and Southeastern Asia. We suggest improving land governance and urban planning to construct eco-friendly landscape structures to mitigate biodiversity loss due to urbanization.

## Introduction

Our world is experiencing a large increase in the urban population. The urban human population proliferated from 1 billion in 1960 to 2 billion in 1986^1^. After 2007, more than half of humanity (4.2 billion in 2018) lived in cities^2^. The dramatic increase in population in cities manifests a fast urbanization process. However, the rapid urbanization process has led to a direct and indirect loss of natural habitat that has re-shaped the pattern of biodiversity^3–7^. On the one hand, based on the projection of future urban expansion by 2050^8^, up to 855 species are directly impacted by urbanization^7^. On the other hand, urbanization may lead to a knock-on effect that compensates for the conversion of cropland into urban land via cropland displacement^6^. Cropland displacement, which means the conversion of natural habitat to cropland, results in indirect habitat loss that is 5 to 10 times greater than habitat loss due to direct urban encroachment^6^. The direct and indirect habitat loss vary among different biogeographic realms. As such, it is essential to overview how urbanization-driven land use/land cover (LU/LC) changes govern biodiversity patterns across biogeographic realms to improve our understanding of community ecology and biogeography.

The majority of urbanization-driven LU/LC change is shifting from Europe and North America to Asia and Africa^1^. However, most broad-scale urban biodiversity studies focused on cities in Europe and North America^9–14^. These studies revealed an increasing focus on incorporating landscape structure (composition and configuration) into explaining biodiversity variation in cities. Aronson et al.^11^ indicated that the proportion of urban land cover in a city is one of the major drivers for explaining plant and bird diversity in more than a hundred cities. On the other hand, some studies went a step further by considering landscape configuration. Turrini and Knop^13^ identified that sufficient vegetated space and habitat connectivity could help mitigate the negative consequences of urbanization on arthropod diversity. Benind et al.^12^ indicated that patch area and corridors had the strongest positive effects on urban biodiversity among 75 cities, compared with the other habitat factors at local (e.g. microclimate, water body structure, and habitat edge, etc.) or landscape levels (e.g. distance to water body and agricultural areas, etc.). These studies documented important landscape features for maintaining biodiversity. Nevertheless, the influences of LU/LC changes on taxa in different biogeographic realms remain unclear. The vertebrate fauna in different biogeographic realms exhibits differences due to various ecological, historical, and geographical factors. Each biogeographic realm represents a distinct geographic region with its unique assemblage of species and ecological characteristics. We still need to explore the consequences in regions predicted to have the greatest urbanization threats to species, such as sub-Saharan Africa, South America, Mesoamerica, and Southeast Asia^7^, and the fastest-growing areas, such as China and India^8^. For developing appropriate urban planning strategies to mitigate biodiversity loss in these regions, it is essential to apply a broad-scale landscape ecological analysis to understand the role of landscape factors in shaping biodiversity patterns^13^.

Understanding the relative importance of different factors in governing biodiversity patterns is a primary goal of community ecology and biogeography^15–17^. A growing body of research has examined how geographical gradients of biodiversity vary due to environmental changes. The environmental factors in explaining biodiversity patterns mainly fall into three categories: energy availability, water availability, and habitat heterogeneity^18,19^. For example, temperature and precipitation were used as a surrogate of pure energy and water availability for modeling broad-scale vertebrate species richness, respectively^18,20,21^. The other studies used elevation range (topographic variability) to measure habitat heterogeneity^22,23^. Nevertheless, LU/LC changes driven by urbanization can be influential as it redistributes suitable habitats in quantity and quality^11^. As such, comparing landscape factors with other factors may provide background information to examine the relative importance of urbanization in explaining variation in species richness.

The central goal of this study is to explore the role of landscape factors in shaping biodiversity in urban areas across biogeographic realms. Using the variation partitioning approach, we quantified and compared the relative contribution of landscape factors (i.e., landscape composition and configuration) in explaining the species richness of three vertebrate classes with environmental factors relevant to water and energy availability. We analyzed 505 urban landscapes with more than one million inhabitants in 2016 at global, regional (biogeographic realms), and country-level extents. By doing so, we provide a landscape ecological perspective for mitigating immediate and profound ongoing human-driven biotic and abiotic impact across various biogeographical realms.

## Results

### The geography of vertebrate diversity in landscapes with more than one million inhabitants

**Figure 1:**
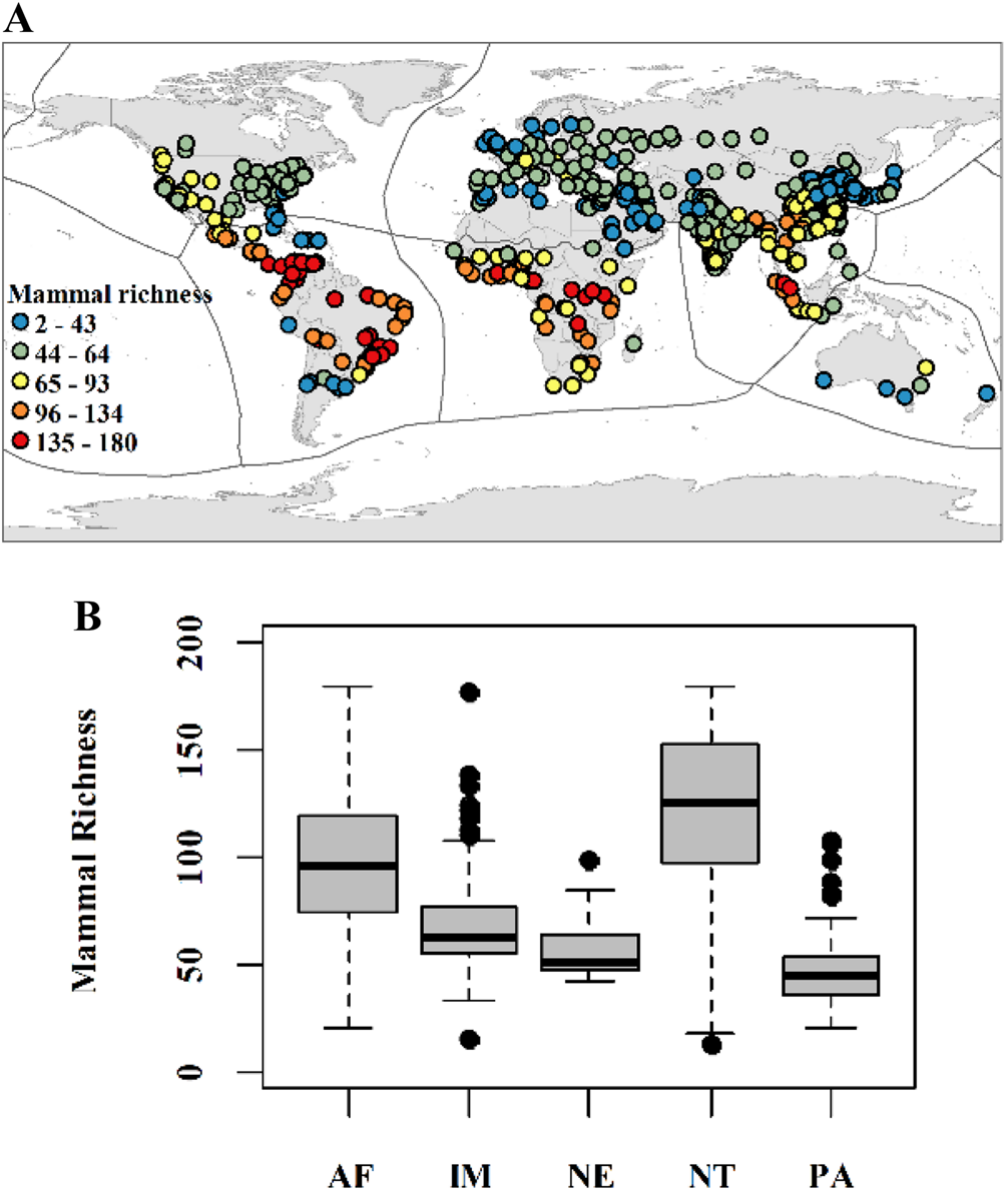
Mammalian species richness: (**a**) selected urban areas and (**b**) a boxplot for the Afrotropical (AF), Indo-Malayan (IM), Nearctic (NE), Neotropical (NT), and Palearctic (PA) realms. Generated with QGIS (3.323).

**Figure 2:**
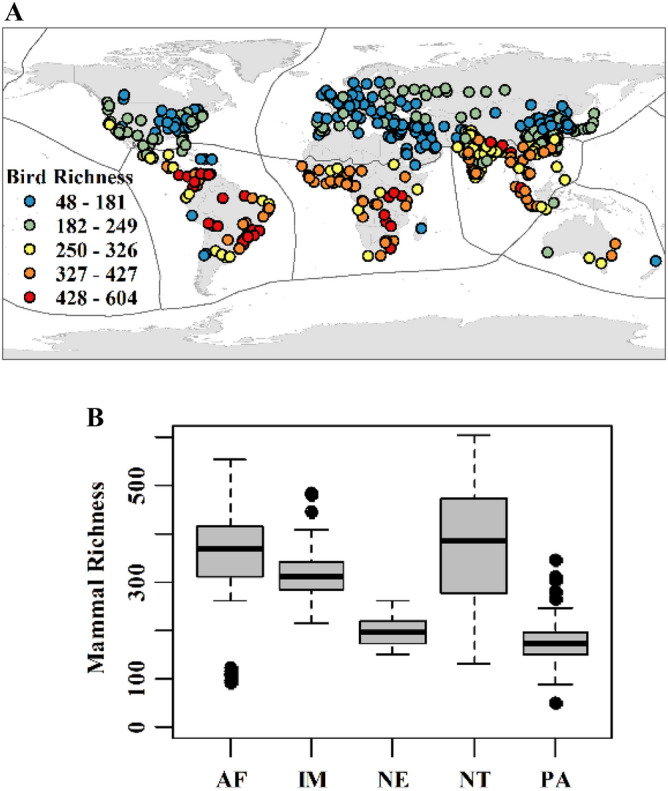
Bird species richness: (**a**) selected urban areas and (**b**) boxplot for the Afrotropical (AF), Indo-Malayan (IM), Nearctic (NE), Neotropical (NT), and Palearctic (PA) realms. Generated with QGIS (3.323).

**Figure 3:**
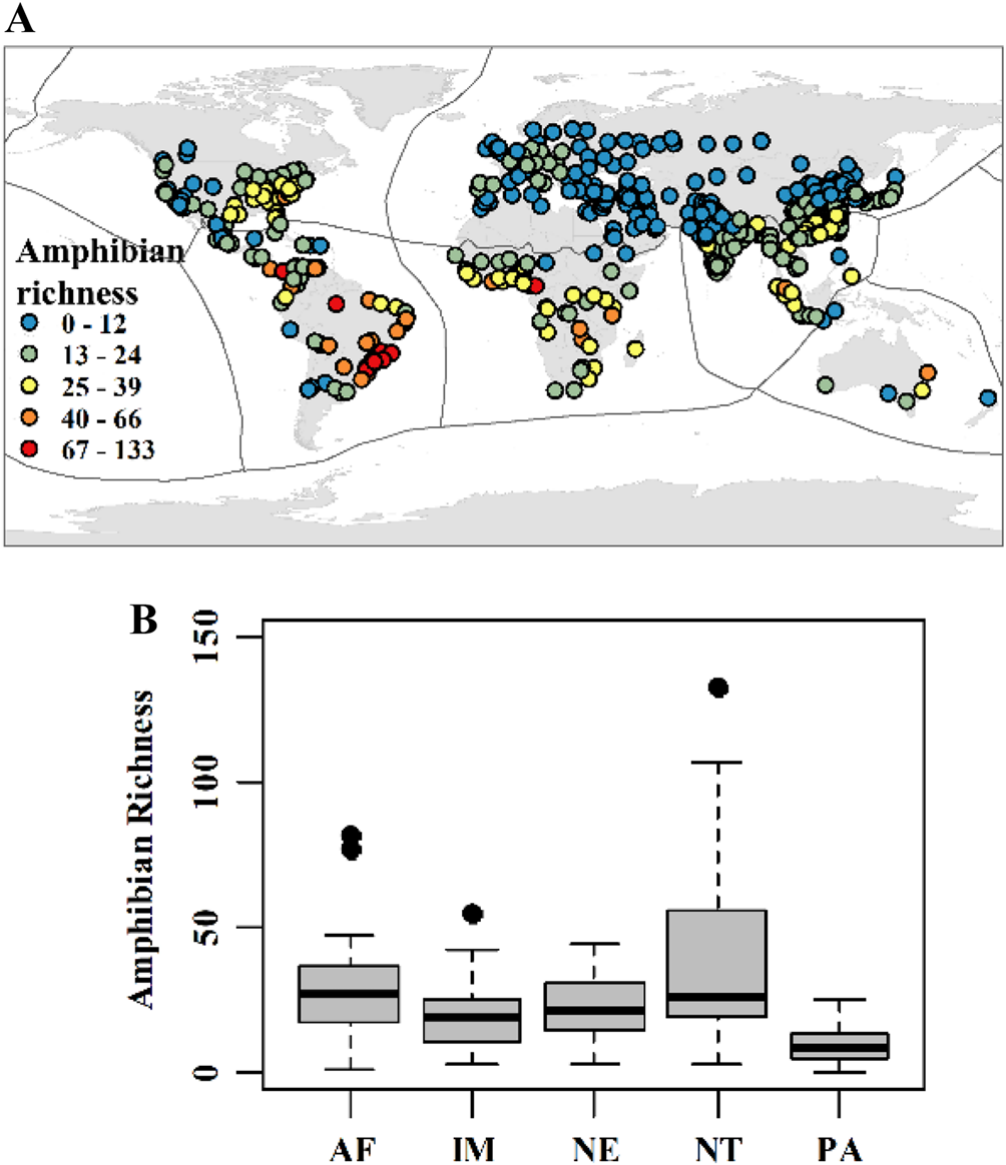
Amphibian species richness: (**a**) selected urban area and (**b**) boxplot for the Afrotropical (AF), Indo-Malayan (IM), Nearctic (NE), Neotropical (NT), and Palearctic (PA) realms. Generated with QGIS (3.323).

We explored patterns of vertebrate species richness within the boundaries of cities that inhabited more than one million people in 2016^24^. The species richness of mammals, birds, and amphibians in these targeted urban landscapes varied among different biogeographic realms (Figs. 1, 2 and 3). The results indicated the highest median of species richness in the Neotropical for all three taxa and the lowest in the Palearctic realm (avian and amphibian) and Australasian realm (mammal). On the other hand, ten and eight high outliers of mammal species richness were found in the Indo-Malayan (124 selected urban landscapes) and Palearctic (202 selected urban landscapes) realms, respectively. Seven high and seven low outliers than the arithmetic average of avian species richness were identified in the Palearctic and Afrotropical (53 selected urban landscapes) realms, respectively. Finally, two, one, and one high outliers in the Afrotropical, Neotropical, and Indo-Malayan realms, respectively.

### Contribution of landscape and environmental factors to the variation of vertebrate species richness

**Figure 4:**
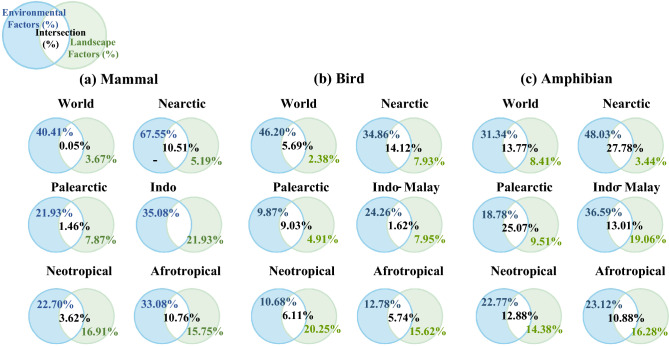
The results of variation partitioning across different regions. (**a**) The variation partitioning explained by environmental and landscape factors for mammals. (**b**) The variation partitioning explained by environmental and landscape factors for birds. (**c**) The variation partitioning explained by environmental and landscape factors for birds. Generated with Microsoft Visio 2016.

Variation partitioning showed that the environmental factors, including climate, topography, and latitude, have the most prominent effect on the species richness of three vertebrate classes globally, the five biogeographic realms, and China and India (for the results of China and India, please see Appendix A), compared with the landscape factors (i.e. composition and configuration) (Fig. 4). Our variation partitioning analysis quantified the contributions of landscape and environmental factors to species richness variation. The results revealed the proportion of explained variation attributed to landscape factors, environmental factors, and their interaction.

**Figure 5:**
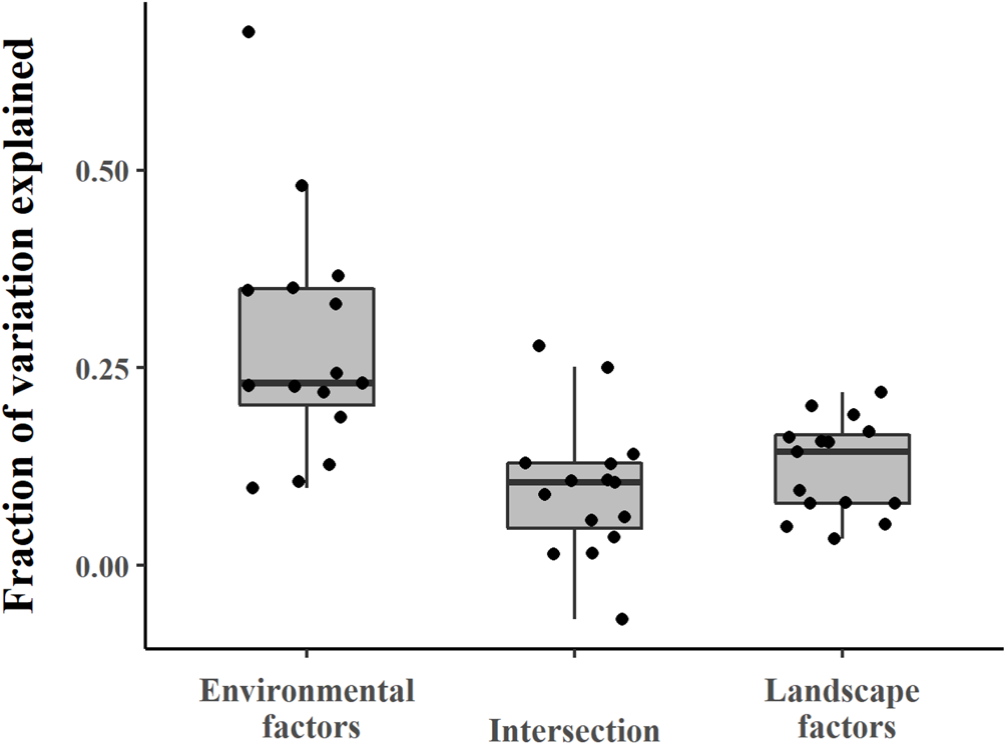
The variation in the species richness of the three vertebrate groups: the explanatory power of the environmental factors, landscape factors, and the intersection between the environmental and landscape factors. Generated with R (4.2.2) and ggplot (3.4.2).

In comparison to the fraction of species richness variation explained only by the environmental factors across the five geographic realms, the Nearctic realm exhibited the greatest influence of environmental factors in explaining the variation in species richness across all three vertebrate classes. On the other hand, the Indo-Malayan realm had the highest fraction of species richness variation explained by landscape factors for mammals and amphibians, while the Neotropical realm had the highest fraction for birds (Fig. 4). Overall, the variance of three vertebrate species richness can be appreciably explained by the environmental factors (an average of 28.14%), which is significantly higher than that of landscape factors (an average of 12.47%) (*P* < 0.001 in ANOVA with pairwise t-test) in urban landscapes for the five biogeographic realms (Fig. 5).

**Figure 6:**
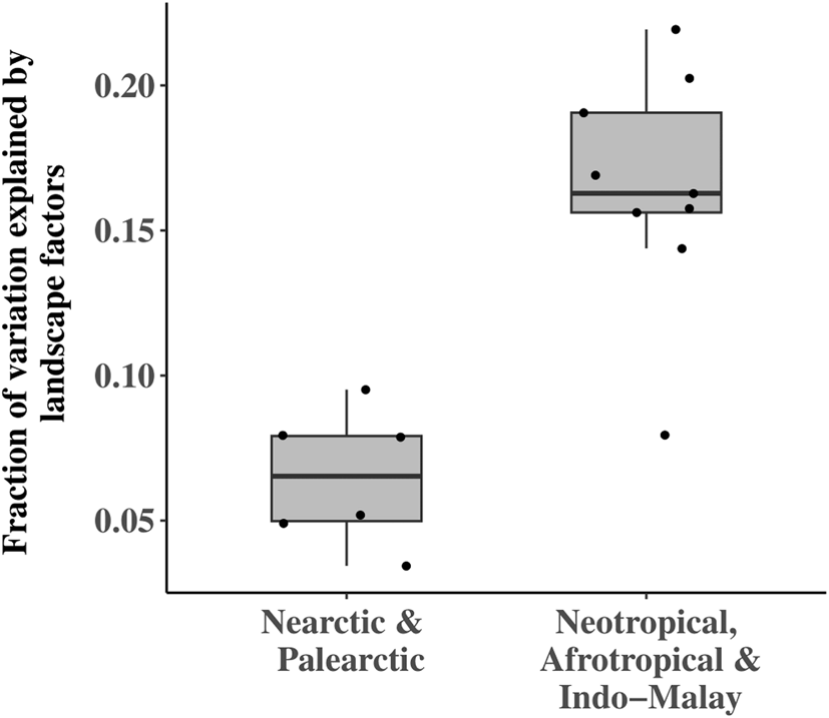
The comparison of the explanatory power of the landscape factors on the three vertebrate taxa richness across different regions. Generated with R (4.2.2) and ggplot (3.4.2).

Our results also showed that the variation of vertebrate species richness explained by landscape factors in the Indo-Malayan, Afrotropical, and Neotropical realms (an average of 16.46% among the three vertebrate classes) is significantly higher than that of the Palearctic and Nearctic realms (an average of 6.10%) (*P* < 0.001 and *P* = 0.041 in ANOVA with pairwise t-test, respectively) (Fig. 6).

### The influential landscape factors of vertebrate diversity in different biogeographic realms

The capability of predictors in explaining variation in vertebrate species richness varied across different regions. Here, we emphasized the influence of landscape factors in Indo-Malayan, Afrotropical, and Neotropical realms (Tables 1, 2 and 3) (see Tables S1 to S3 for the other regions). In these regions, class area of forest (CA_*Forest*_) was the most selected landscape factor positively affecting the diversity of three vertebrate classes. For example, CA_*Forest*_ positively affects mammal diversity in Indo-Malayan realm, bird diversity in Neotropical realm, as well as amphibian diversity in Neotropical and Indo-Malayan realms. On the other hand, CA_*Wetland*_ positively affects all three vertebrate diversity in China. However, the vertebrate diversity was increased with CA_*Built up*_ in Afrotropical (three vertebrate classes) and Neotropical realms (mammal species).

**Table 1:** The best subset selection of the Poisson regression model for mammal diversity in urban landscapes with at least a million inhabitants.

	Mammals	Neotropical	Afrotropical	Indo-Malay
	(Intercept)	4.9945***	4.5058***	5.2747***
Environmental factor	Latitude	− 1.3755***	− 1.0492***	–
	Mean elevation	–	0.3056•	− 0.7709***
	CV of elevation^a^	–	–	–
	Mean temperature	–	–	− 1.0295***
	Temperature seasonality	–	–	− 0.9186***
	Precipitation seasonality	–	− 0.3842•	–
Landscape factors	CA_*Forest*_	–	–	0.4582***
	CA_*Wetland*_	− 1.4056•	–	–
	CA_*Built up*_	1.6646**	0.5386**	–
	Coh_*Forest*_	0.7767*	–	–
	Coh_*Built up*_	− 0.6615 •	–	0.22778
	ED_*Forest*_	–	0.4094	–
	ED_*Wetland*_	–	–	− 1.1968***
	ED_*Built up*_	− 0.5482•	0.4126*	–
	* R*_*adj*_^*2*^	0.4323	0.5959	0.5020

Significant differences: ^•^*P* < 0.1, *P < 0.05, **P < 0.01, and ***P < 0.001.

^a^Coefficient of variation of elevation.

**Table 2:** The best subset selection of the Poisson regression model for bird diversity in urban landscapes with at least a million inhabitants.

	Birds	Neotropical	Afrotropical	Indo-Malay
	(Intercept)	5.7865***	5.6291***	6.2639**
Environmental factor	Latitude	− 0.4487**	− 0.8832***	–
	Mean elevation	–	–	–
	CV of elevation^a^	–	–	− 0.3505***
	Mean temperature	–	–	− 0.6220***
	Temperature seasonality	–	–	–0.4243***
	Mean Precipitation	–	–	0.1265
	Precipitation seasonality	− 0.4733*	–	0.2201**
Landscape factors	CA_*Forest*_	0.5078**	–	–
	CA_*Wetland*_	–	–	–
	CA_*Built up*_	–	0.5044*	–
	Coh_*Forest*_	0.4903*	–	–
	Coh_*Built up*_	–	–	–
	ED_*Forest*_	–	0.6935**	0.1108 •
	ED_*Wetland*_	–	–	− 0.2372*
	ED_*Built up*_	–	0.5039*	–
	ES_*Forest, Built up*_	–	–	0.2393**
	* R*_*adj*_^*2*^	0.3704	0.4119	0.3384

Significant differences: ^•^*P* < 0.1, *P < 0.05, **P < 0.01, and ***P < 0.001.

^a^Coefficient of variation of elevation.

**Table 3:** The best subset selection of the Poisson regression model for amphibian diversity in urban landscapes with at least a million inhabitants.

	Amphibians	Neotropical	Afrotropical	Indo-Malay
	(Intercept)	2.2439***	2.2739***	3.9102***
Environmental factor	Latitude	–	− 1.2444**	–
	Mean elevation	–	–	− 0.9208**
	CV of elevation^a^	–	–	–
	Mean temperature	–	–	− 0.9864*
	Temperature seasonality	–	–	− 0.7715**
	Mean precipitation	2.0273***	1.1284*	0.4448•
	Precipitation seasonality		–	− 0.4158*
Landscape factors	CA_*Forest*_	0.8917*	–	0.5791***
	CA_*Wetland*_	–	–	–
	CA_*Built up*_	–	0.8310*	–
	Coh_*Forest*_	–	0.6324•	0.2789*
	Coh_*Wetland*_	–	–	–
	Coh_*Built up*_	–	–	–
	ED_*Forest*_	–	0.8422*	–
	ED_*Wetland*_	–	–	− 1.6200***
	ED_*Built up*_	–	–	–
	ES_*Forest, Built up*_	0.7531•	–	–
	* R*_*adj*_^*2*^	0.5003	0.5028	0.6866

Significant differences: ^•^*P* < 0.1, *P < 0.05, **P < 0.01, and ***P < 0.001.

^a^Coefficient of variation of elevation.

The cohesion of forest (Coh_*Forest*_) increased mammal and bird species richness in the Neotropical realm and amphibian species richness in the Indo-Malayan realm. On the other hand, the positive influence of Coh_*Built up*_ reflects that landscapes with aggregated built-up areas had a higher species richness of the three vertebrate communities in the Afrotropical realm.

Edge density is the measure of the total length of edges, such as habitat boundaries or forest edges, in relation to the total area of a landscape. The edge density of a specific land-use type reflects the number of edge habitats. While the cohesion index captures the overall connectivity and fragmentation of the landscape, the edge density measures the amount and extent of habitat edges within the landscape. The edge density of the forest positively influenced bird and amphibian species richness in the Afrotropical realms. On the other hand, the diversity of the three vertebrate classes decreased when the edge density of wetland increased in the Indo-Malayan realm.

## Discussion

### Landscape composition and configuration are important in biogeographic realms experiencing rapid urban growth

Our study reveals the importance of landscape structure (i.e. composition and configuration) on the variation of mammal, avian, and amphibian species richness in landscapes with at least one million inhabitants across biogeographic realms. Although the variation of vertebrate species richness can be better explained by environmental factors (i.e. climate, latitude, and elevation) than the landscape factors, we pointed out the higher impact of landscape structure in shaping biodiversity in the Afrotropical, Neotropical, and Indo-Malayan realms, compared to the other realms. More importantly, these realms overlap with regions that are expected to face the most significant threat from urbanization to species by 2050, as projected by Simkin et al.^7^. The regions with high predicted urbanization impact on biodiversity include sub-Saharan Africa, South America, Mesoamerica, and Southeast Asia^7^. In other words, our finding suggests that the unique species assemblages in each of these realms, which are experiencing an unprecedented rate and magnitude of urbanization^1,3,7^, are particularly sensitive to landscape alterations. As such, mitigating the impact of urbanization on biodiversity requires attention to land-use planning in these regions. Nevertheless, many countries in these fast-growing regions have lower land governance than other regions^5^.

Similar to the influences of the environmental factors, the unique ecological history and evolution of each biogeographic realm can be shaped by the landscape factors at different levels. For example, Rowan et al.^17^ indicated that human-driven land-use changes significantly affected the mammalian communities in the Indo-Malayan and Neotropical realms rather than the other regions. In contrast to previous studies on urban biodiversity centered primarily on Europe and North America^9–14^, we provide more comprehensive information to recognize influential drivers on urban biodiversity variation in the fast-growing regions. Moreover, based on the analysis of landscapes with more than one million inhabitants, our findings provide clues regarding the composition and configuration of urban landscapes that benefit the three vertebrate taxa (mammals, birds, and amphibians) for urban planning in developing cities within the fast-growing regions.

### Conservation strategies based on the important landscape factors

Our study not only prioritizes regions where international aid for conservation is urgently needed to mitigate urbanization impact but also highlights the influential land-use patterns that contribute to biodiversity variation, providing valuable insights for urban planners and local practitioners in developing conservation strategies. International aid refers to various forms of conservation support provided by international organizations or countries, including financial assistance, capacity building, and training aimed at protecting wildlife. On the one hand, international stakeholders, such as non-governmental organizations and multilateral funding agencies, can redirect their conservation efforts towards preserving biodiversity areas near developing cities in the Afrotropical, Neotropical, and Indo-Malayan realms. On the other hand, our study provides influential land-use patterns on the three vertebrate classes for land-use and urban planners. The landscape ecological views for allocating land-use patterns have been widely integrated as mitigation strategies into urban planning. For example, to mitigate urbanization impact on the California cougar (*Felis concolor californicus*), landscape planners created steppingstones or connected habitats between San Diego and Los Angeles (i.e. Camp Pendleton Region)^25^.

### The influence of landscape composition on the fast-growing regions

The landscape ecological analysis provides information for planning rapidly developing cities in fast-growing regions. Our findings denote a positive influence of forest area on vertebrate diversity in China, India, the Neotropical, and the Indo-Malayan realm. Previous studies have reinforced the importance of maintaining forest cover in mitigating the impacts of urbanization on mammals, such as the Asian elephant (*Elephas maximus*) in Southeastern Asia^26^, Chinese pangolin (*Manis pentadactyla*) in China^27^, as well as amphibians, such as Darwin’s frog (*Rhinoderma darwinii*) in South America^28^ and Galaxy Frog (*Melanobatrachus indicus*) in India.

Maintaining forest land cover is the key priority in addressing land use planning for these fast-growing regions. Two examples of urban planning illustrate the policy instruments to maintain forest land cover, either by establishing a protected area or an urban greenspace network, in cities within the Indo-Malayan realm. The Malaysian government established forest reserves (e.g. Ulu Langat and Ampang Water Catchment Forest) within the metropolitan area of Kuala Lumpur. On the other hand, Singapore implemented policies that promote green interventions by establishing a network of greenspaces as an alternative approach to maintaining urban biodiversity^29^. On the other hand, the positive influence of class area of wetlands in China is consistent with the causal effect of wetland conservation on vascular plant and vertebrate diversity in 186 natural reserves, as revealed by Zhao et al.^30^ However, the vertebrate diversity in the Afrotropical and Neotropical realms may increase with built-up areas due to the fact that urbanization occurred around the protected areas^31^.

### The influence of landscape configuration on the fast-growing regions

Other influential landscape factors reveal suitable landscape configurations (i.e. spatial patterns) for preserving biodiversity. The connectivity of forests facilitates species movement and gene flow to maintain functional connectivity for ecological integrity. However, 70% of the remaining global forest located within 1 km of the forest edge is at risk of fragmentation, resulting in biodiversity reductions ranging from 13 to 75%^32^. Our findings indicate that the cohesion of forest, which reflects the connectivity of forest, positively influences mammalian and avian communities in the Neotropical realm. Similar to our results, the loss of forest connectivity has been reported to negatively affect species in the Neotropical realm, such as jaguars (*Panthera onca*)^33^ and understory avian communities^34^ in the Atlantic Forest, which extends across the eastern coast of Brazil, Argentina, and Paraguay in South America.

On the other hand, edge density focuses on the amount of edge habitat or the length of habitat edges within a landscape. However, forest edges significantly affect over half of the forests globally, leading to a worldwide decline in biodiversity^35^. Edges between forest and non-forest habitats result in ecological effects that alter the biophysical environments for species. The edge effect can be complementary to habitat fragmentation. Previous studies indicated the negative impact of forest edges on forest-core species. These forest-core species were more likely listed as threatened species by the International Union for Conservation of Nature (IUCN), such as Baird’s tapir (*Tapirus bairdii*) and the Bahia tapaculo (*Eleoscytalopus psychopompus*)^35^. Moreover, the synergy effect with subsistence hunting on Amazonia forest vertebrates can amplify the edge effects due to rainforest fragmentation^36^. As such, fragmented forest landscapes increase the possibility of overexploitation on midsized to large vertebrate species. In addition to analyzing forest edges, we indicate that avoiding the increase of wetland edges is essential for all three vertebrate classes in the Indo-Malayan realm. Nevertheless, there still needs to be more research on the impact of wetland edges on vertebrate diversity in this region.

Despite the fact that forest fragmentation often causes biodiversity loss, reactions of species may be complex and inconsistent^37,38^. The edge effect may adversely affect species that prefer the forest core while improving habitat suitability for species that prefer the edge or between the core and the edge^35,37,39–41^. Our findings suggest that forest edge density may improve birds and amphibian diversity in the Afrotropical realm. For amphibian species, previous studies reported the highest diversity at the edges of fragmented forests in Madagascar^37,40^. The phenomenon resulted from the increase of shared species between forest and edge habitat (i.e. generalists) in accordance with the presence of streams at the edges^37,40^.

For bird species, previous studies reported negative influences of forest edge that decrease avian diversity in Africa^38,42,43^. However, we argue that contradictory findings may result from differences in targeted urban landscapes. Our findings result from partially urbanized or urbanizing landscapes, such as Kigali in Rwanda, Kampala in Uganda, and Mombasa, Nairobi in Kenya. The remnant forests that benefit bird or amphibian species are fragmented in these landscapes. In contrast, the aforementioned edge effect studies focused on the tropical rainforests in the Afrotropical realm. Morton et al.^38^ reported lower species richness in the forest fragment in Nyungwe National Park, Rwanda, covered by tropical montane forests. Dale et al.^43^ reported a significant increase in the number of species with distance from the edge of a tropical forest in Budongo Forest Reserve, Uganda. Mammides et al.^42^ indicated an edge effect on all bird categories except on forest visitors in Kakamega Forest, Kenya. These studies reveal the complexity of edge effects on species shaped by habitat types, landscape configuration, and species life history (e.g. reproductive strategy, age at maturity, lifespan, fecundity, parental care, and growth rate). For example, Morton et al.^38^ indicated that the population size of bird species in Nyungwe National Park, Rwanda was also influenced by intrinsic factors, such as body size and life history^38^. As such, the complexity of the turnover between forest-core and forest-edge preferring species needs to be considered in either species (e.g. selection of targeting species) or ecosystem (e.g. selection of suitable landscapes to be protected) approach for developing suitable conservation strategies against urbanization.

### The influence of environmental factors in the fast-growing regions

Our study not only indicated important landscape factors but also environmental factors, such as climate, elevation, and latitude. However, compared to the previous studies^18,19,21,23^, similarity of the environmental constraints exists in the urban landscapes. For example, Barreto et al.^19^ found temperature and precipitation are generally strong predictors in explaining biodiversity gradient of terrestrial tetrapods. Our results also revealed the influence of temperature and precipitation on mammal and amphibian communities at different levels across urban landscapes in different regions. On the other hand, Qian^18^ revealed that the environmental constraints to ectotherms might be responsible to higher beta diversity for amphibians than for mammals and birds. Our results of variation partitioning showed similar results in the urban landscapes in the Neotropical, Afrotropical, and Indo-Malayan realms. In these regions, fraction of biodiversity variation explained by the environmental factors is higher for amphibians than mammals and birds, besides mammals in the Afrotropical realm.

The other studies mainly focused on the influences on avian taxa. Pigot et al.^21^ found that key climatic variables, such as temperature, precipitation, and topography, can explain spatial variation in range shape of birds. Davies et al.^23^ indicated that topographical variability and temperature are the most important global predictors of avian species richness. Similar to these results, our results indicated that a variety of temperature, mean and variety of elevation are significant to bird species richness of urban landscape worldwide.

### Limitations and future considerations

By 2030, urbanization is expected to occur in high-biodiversity areas with weak land governance^5^. It is estimated that 13% of terrestrial endemics will be in ecoregions under the highly predicted impact of urban-caused habitat conversion^44^. However, addressing worldwide species checklists of urban landscapes, which are spanning urban–rural gradients requires countless individual effort and time. At this moment, we advocate for an urgent macro-scale landscape ecological analysis to explore the interplay between landscape patterns and terrestrial biodiversity. Although we indicated consistent results aligning with findings from local studies, such as influences of forest area and edge on vertebrate communities, our study could only provide insights for establishing an initial baseline. The fine-resolution species checklists of large and densely populated urban landscapes are needed be addressed in the future studies. With the availability of more comprehensive data in the future, it will enable more extensive comparisons. Moreover, we suggest that future studies need to focus on the urban landscape configuration of cities near protected areas or areas with high biodiversity across continents.

On the other hand, comparing the influences of environmental factors and landscape factors on vertebrate diversity may be spatial scale-dependent. The impact of human-driven land use and land cover changes on vertebrate species richness might become more influential as the scale shifts from coarse (regional) to fine (local)^45^. For example, Heino et al.^46^ indicated that urban land use could better explain the diversity of ground beetles than the annual temperature at a regional scale than at a global scale. Future studies can go a step further to compare the impact of land-use factors on biodiversity with environmental factors from global to local scales across different biogeographic realms. As such, comprehensive information can be provided to country or local-level conservation practices. Moreover, future studies need to expand the research scope to encompass a more comprehensive array of countries and regions.

Our study only selected a few landscape metrics for representing the landscape factors. Future studies could incorporate more types of landscape metrics for developing suitable urban planning strategies to address biodiversity threats due to urbanization.

## Materials and methods

### Cities with more than one million inhabitants

**Figure 7:**
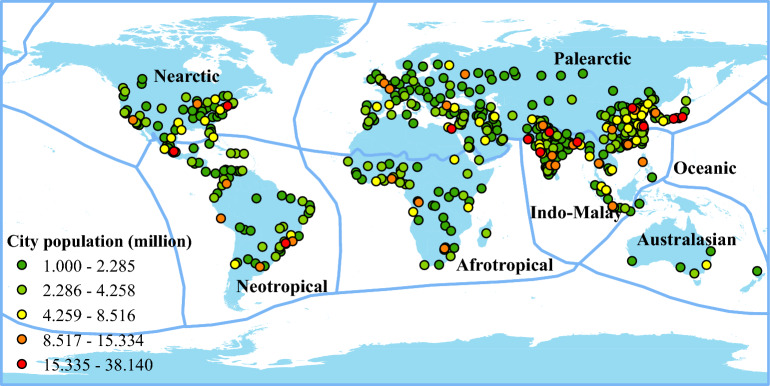
Cities with at least one million inhabitants in 2016 across biogeographic realms. Generated with QGIS (3.323).

This study analyzed the relationship between landscape structures with vertebrate species richness for 505 cities with more than one million inhabitants in 2016^24^ (Fig. 7) in six biogeographic realms^47^. These cities have a wide range of boundary definitions. The boundary definitions consist of city proper, urban agglomeration, and metropolitan area^24^. The city proper indicates the administrative boundary of a city. Urban agglomeration refers to the extent of the contiguous built-up area. Finally, the metropolitan area defines its boundary based on interlinked commerce or urban commuting patterns. The definition of the three urban boundaries does not represent an absolute order of size. The area size of an urban area defined by the city proper depends on the urban policies of a country. For some urban areas, the city proper can be larger than the extent of the contiguous built-up areas. The area of the 505 target cities ranges from 4194 to 350,811 ha, with a mean area of approximately 299,040 hectares.

All 505 city boundaries covered a landscape with an embedded urban area. The extent of a landscape with an embedded city conforms to a landscape-level urban biodiversity study^48^. Our study analyzed the relationship between vertebrate species richness and landscape structures covering a landscape mosaic along an urbanization gradient, rather than focusing solely on an urban matrix. We explored the relationships at a global scale, encompassing biogeographic realms as well as specific regions such as China and India. China and India were predicted to have the highest urbanization growth rates^3,8^. Nevertheless, the Australasian realm comprises only seven selected cities (i.e. 7 samples), which is insufficient to perform best subset selection to address predictors selection (up to 18 candidates) for the Poisson regression individually. See Appendix C for the estimation of total sample size based on the power analysis using G*Power software^49^.

### Vertebrate species richness and factors limiting species’ ranges for identifying the response of broad-scale urban biodiversity patterns

To compare the influences of landscape factors with other environmental factors, we collected data on vertebrate species, land coverage, climate, elevation, and latitude for the targeted 505 cities. First, based on the boundaries of each city, we extracted the representation of vertebrate species richness from a global raster dataset of mammal, bird, and amphibian species richness complied by Jenkins et al.^50^ in 2013. Urbanization can impact animal movement patterns (i.e. home ranges), due to factors like habitat fragmentation, landscape barriers, and resource density and availability^51^. As such, we selected three vertebrate classes to investigate the influence of landscape composition/configuration resulting from past or ongoing urbanization.

For the predictors that reflect landscape composition and configuration, we quantified landscape metrics for the targeted urban landscapes, using land use data in 2010 at the resolution of 30 m from GLOBELAND30^52^. On the other hand, for the environmental factors that consist of climatic and altitudinal differences between the targeted urban landscapes, we used bioclimatic variables, including annual mean and seasonality of temperature and precipitation from 1970 to 2000^53^. Mean and variation of elevation were compiled from ALOS World 3D-30m (AW3D30)^54^.

### Variation partitioning

This study estimated the unique and shared contributions of landscape and environmental factors in explaining different patterns of vertebrate species richness using the variation partitioning approach^55–57^. The variation partitioning approach is an extension of multivariate regression. Variation partitioning (*Y* ~ *X*) indicates that variation in a responsible variable Y can be partitioned into fractions, including variation explained by each set of predictors *X*_1_, *X*_2_, …., *X*_*n*_ (each represents a predictor matrix), the interaction between the predictor matrices, and unexplained variation^57^. The contribution of each predictor matrix and their interaction can be evaluated by adjusted *R*^2^ (*R*^2^_*adj*_)^55,56^. Using the approach, we partitioned the total variation in the vertebrate species richness *Y*_*richness*_ into three components: the two unique contributions (landscape factors *X*_*landscape*_ and environmental factors *X*_*environment*_) and the joint contribution of them (*X*_*landscape*_ ∩ *X*_*environment*_). The explanatory variables in the two predictor matrices, *X*_*landscape*_ and *X*_*environment,*_ were selected based on the best model selection results of Poisson regression. We repeated the variation partitioning analysis for the three taxa of vertebrates, namely mammals, birds, and amphibians, across the entire world, different biogeographic realms, China, and India.

### Poisson regression analysis

Understanding the relationship between species richness and environmental explanatory variables plays a significant role in biogeography and ecology studies. This study incorporated landscape factors, using landscape metrics to manifest influences of LULC changes on vertebrate species richness in the targeted urban landscapes. As species richness data are non-negative count data, which are discrete and often heavily right-skewed, we employed Poisson regression to analyze how environmental and landscape explanatory variables affect the species richness of the three vertebrate classes:

1 $$\log (E(Y)) = \beta_{0} + \beta_{1} x_{1} + \cdots + \beta_{i} x_{i} + \varepsilon$$

To avoid multicollinearity among explanatory variables, we deleted the variables with high collinearity in a stepwise manner until the Pearson’s correlation coefficient > 0.7 for all variables excluded in the model. Then, we applied a best subset selection approach that considers all permutations of explanatory variables to address predictor selection^58,59^ for the Poisson regression model before variation partitioning, using *bestglm* package in R^60^.

### Landscape metrics

To quantify the landscape factors (i.e. landscape composition and configuration) of the 505 cities, we used five landscape metrics, including the class area of a specific land use type *l* (*ca*_*l*_), the sum of the edge lengths between two land use types *l* and *m* (*es*_*l,m*_), patch cohesion (*coh*_*l*_), edge density (*ed*_*l*_), and large patch index (*LPI*). The functions were as follows^39,61^:

2 $$ca_{l} = \sum\limits_{p = 1}^{n} {a_{p,l} }$$

3 $$es_{l,m} = \sum\limits_{p = 1}^{n} {ec_{p,l,m} }$$

4 $$coh_{l} = \left[ {1 - \frac{{\sum\nolimits_{p = 1}^{n} {P_{p,l} } }}{{\sum\nolimits_{p = 1}^{n} {P_{p,l} \sqrt {c_{p,l} } } }}} \right]\left[ {1 - \frac{1}{{\sqrt {C_{r} } }}} \right]^{ - 1} \times 100$$

5 $$ed_{l} = \frac{{\sum\nolimits_{m = 1}^{k} {es_{l,m} } }}{A} \times 10000$$

6 $$LPI = \frac{1}{A}\mathop {\max }\limits_{{}} \left( {a_{p,l} } \right) \times 100$$

where *a*_*p,l*_ (m^2^) is the area of patch *p* and land use type *l*. *ec*_*p,l,m*_ (m) is the number of edge cells in patch *p* between land use type *l* and land use type *m*. *coh*_*l*_ denotes patch cohesion of land use type *l*. *P*_*p,l*_ is the summation of the perimeters of patches in terms of the number of cell surfaces exposed at the edge of different land-use types. *C*_*p,l*_ is the area of patch *p*. *C*_*r*_ is the total number of cells in the study area. *ed*_*l*_ is the edge density of land use type *l*. *es*_*l,m*_ (m) includes landscape boundary and background edge segment involving land use type *l*. *A* (m^2^) is the total area of a targeted landscape.

### Supplementary Information

Supplementary Information.

## Data Availability

All data generated or analyzed during this study are included in this published article and the Supplementary information.
