# Supplementary material for: The landscape ecological view of vertebrate species richness in urban areas across biogeographic realms
Source: Sci Rep. 2023 Oct 3;13:16647. doi: 10.1038/s41598-023-43896-z (PMC10547837; doi:10.1038/s41598-023-43896-z)
Supplement: Supplementary file 1 — Supplementary Information. [file 41598_2023_43896_MOESM1_ESM.docx]

The landscape ecological view of vertebrate species richness in urban areas across biogeographic realms

Chun-Wei Huang^1,*^, Jia Qing Ooi^2, ⸸^, and Si Ying Yau^2, ⸸^

^1^Ming Chi University, General Education Center, New Taipei City, 243303, Taiwan.
^2^National Taiwan University, Department of Geography, Taipei, 10617 Taiwan.
^*^cwhuang@mail.mcut.edu.tw; chun-wei.huang@aya.yale.edu
^⸸^these authors contributed equally to this work

***Appendix A. The inclusion of China and India in comparison of the influences of landscape factor.***

This study analyzed not only five biogeographic realms but also China and India (Fig S1). The variation of vertebrate species richness explained by landscape factors in China and India (an average of 11.88%) was significantly higher than that of the Palearctic and Nearctic realms (an average of 6.10%) (*P* = 0.041 in ANOVA with pairwise t-test) (Fig S2).

**
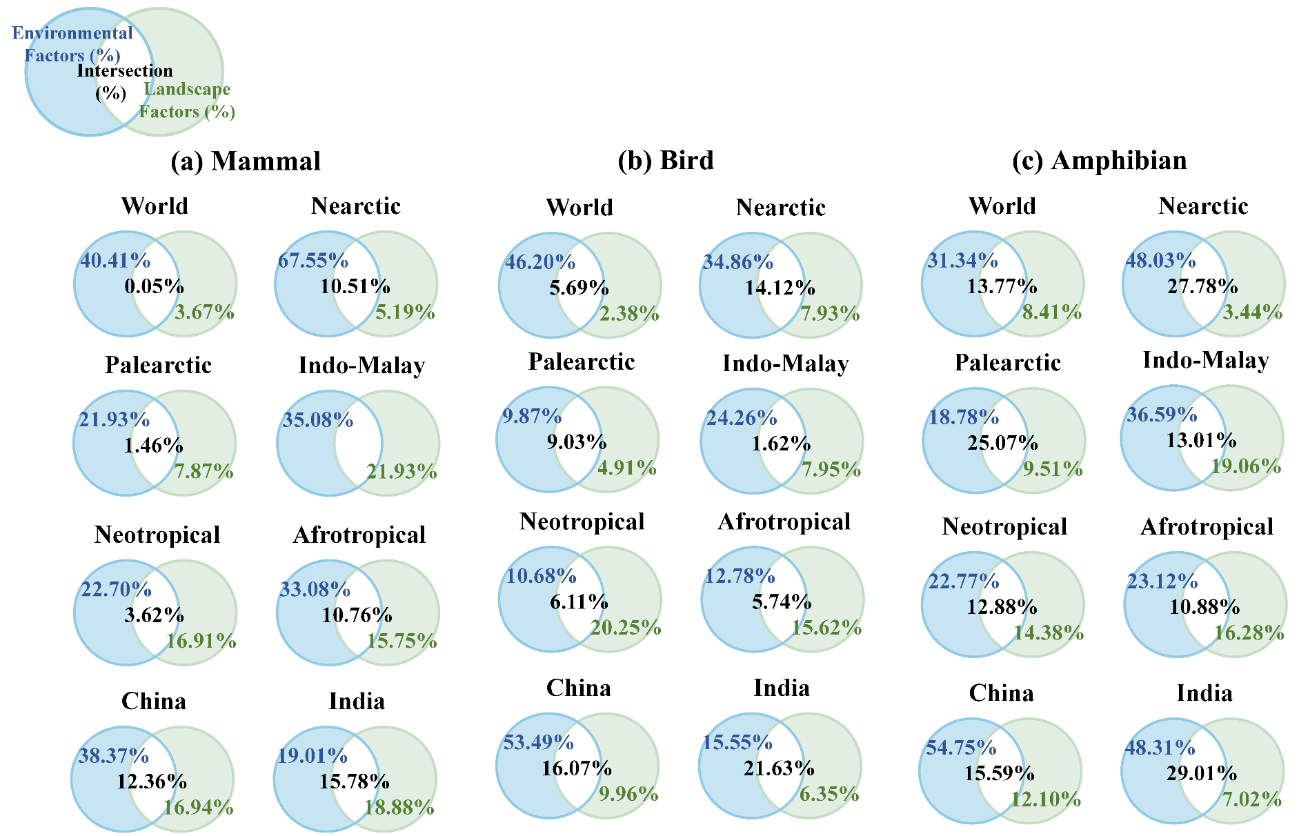
**

**Figure S1. The results of variation partitioning across different regions, including China and India. (a) the variation partitioning explained by environmental and landscape factors for mammals. (b) the variation partitioning explained by environmental and landscape factors for birds. (c) the variation partitioning explained by environmental and landscape factors for birds.**

**
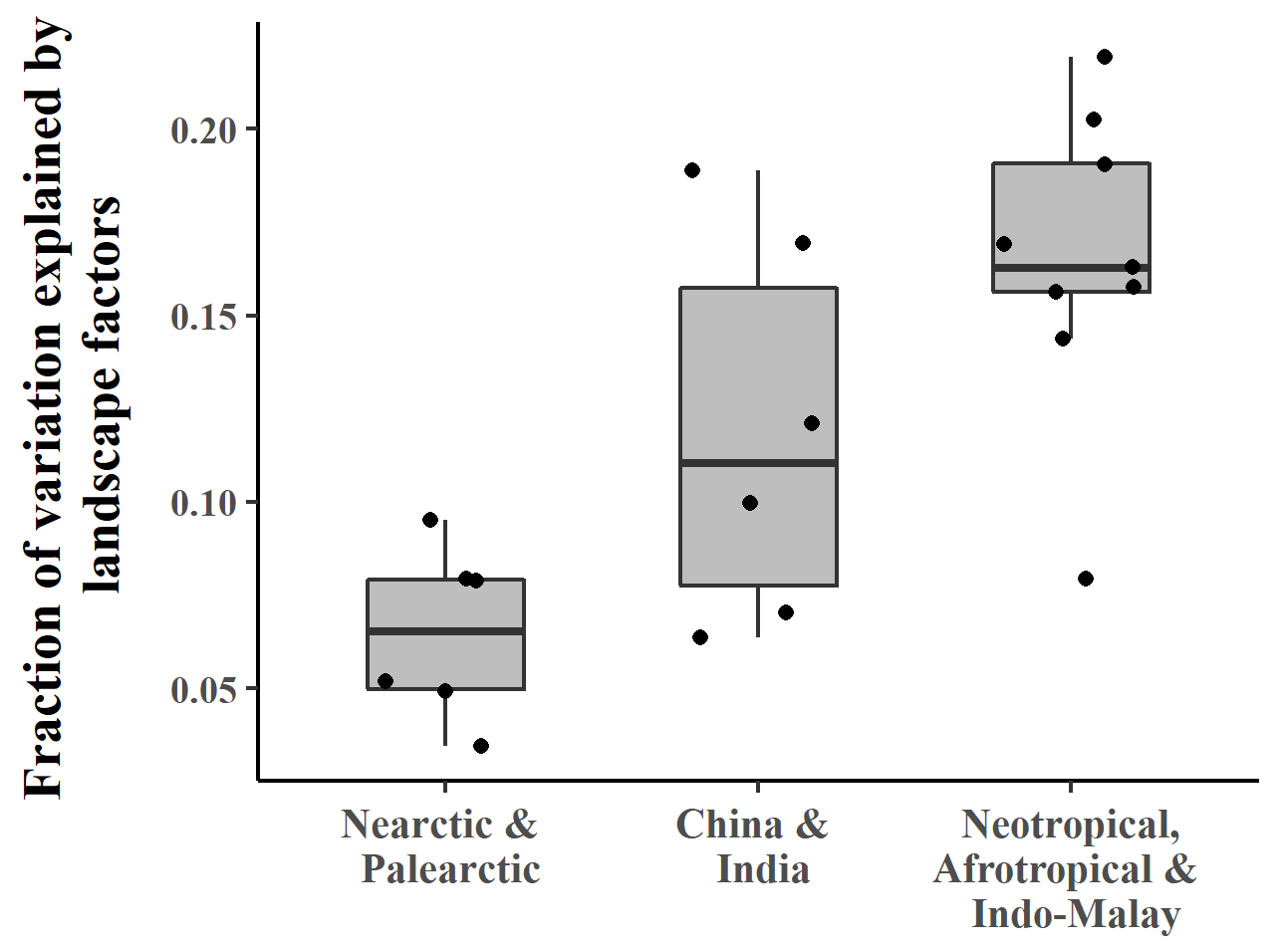
**

**Figure S2. The comparison of the explanatory power of the landscape factors on the three vertebrate taxa richness across different regions, including China and India.**

***Appendix B. Influential determinants in explaining vertebrate richness selected by a best subset selection approach of Poisson regression***

**Table S1. The best subset selection of the Poisson regression model for mammals in different urban regions**

|  | **Mammals** | **World** | **Nearctic** | **Palearctic** | **Neotropical** | **Afrotropical** | **Indo-Malay** | **Australasia^1^** | **China** | **India** |
| --- | --- | --- | --- | --- | --- | --- | --- | --- | --- | --- |
|  | Number of cities | 505 | 58 | 202 | 61 | 53 | 124 | 7 | 104 | 59 |
|  | (Intercept) | 4.2897*** | 3.2757*** | 3.9065*** | 4.9945*** | 4.5058*** | 5.2747*** | - | 4.2719*** | 4.9181*** |
| Environmental factors | Latitude | - | - | - | -1.3755*** | -1.0492*** | - | - | - | -0.5384*** |
|  | Mean Elevation | 0.8923*** | 0.5716*** | 0.6656*** | - | 0.3056 • | -0.7709*** | - | 0.5073*** | - |
|  | CV of Elevation^2^ | -0.4639** | 0.3076*** | - | - | - | - | - | - | -0.3925 • |
|  | Mean Temperature | - | 0.3328*** | - | - | - | -1.0295*** | - | - | -0.8230** |
|  | Temperature Seasonality | -0.9165*** | 0.3319* | - | - | - | -0.9186*** | - | -0.8312*** | - |
|  | Mean Precipitation | 0.6302*** | 0.3187** | - | - | - | - | - | - |  |
|  | Precipitation Seasonality | - | 0.2222* | -0.3860*** | - | -0.3842 • | - | - | -0.2991** | 0..1664 • |
| Landscape factors | CA *_Forest_* | 0.6300*** | - | - | - | - | 0.4582*** | - | 0.3948** | 0.3802*** |
|  | CA *_Wetland_* | -0.4900 | - | - | -1.4056 • | - | - | - | 0.5375** | - |
|  | CA *_Built up_* | - | - | - | 1.6646** | 0.5386** | - | - | - | - |
|  | Coh *_Forest_* | - | - | - | 0.7767* | - | - | - | - | - |
|  | Coh *_Wetland_* | - | - | - |  | - | - | - | - | - |
|  | Coh *_Built up_* | - | - | - | -0.6615 • | - | 0.22778 | - | 0.53747** | - |
|  | ED *_Forest_* | - | - | 0.3709*** | - | 0.4094 |  | - |  | - |
|  | ED *_Wetland_* | - | -0.2746*** | - | - |  | -1.1968*** | - | -0.3990* | - |
|  | ED *_Built up_* | - | -0.1231 | - | -0.5482 • | 0.4126* | - | - | -0.2767* | - |
|  | ES *_Forest, Built up_* | - | - | - | - | - | - | - | - | - |
|  | Large Patch Index (LPI) | -0.3669*** | 0.0816 | -0.2207* | - | - | - | - | - | - |
|  | *R_adj_^2^* | 0.4413 | 0.8326 | 0.3126 | 0.4323 | 0.5959 | 0.5020 | - | 0.6768 | 0.5367 |

Significant differences : ^•^ P<0.1, *P<0.05, **P<0.01, and ***P<0.001 .

^1^ The sample size of the Australasian realm is not statistically representative.

^2^ Coefficient of variation of elevation.

**Table S2. The best subset selection of the Poisson regression model for birds in different urban regions**

|  | **Birds** | **World** | **Nearctic** | **Palearctic** | **Neotropical** | **Afrotropical** | **Indo-Malay** | **Australasia^1^** | **China** | **India** |
| --- | --- | --- | --- | --- | --- | --- | --- | --- | --- | --- |
|  | Number of cities | 505 | 58 | 202 | 61 | 53 | 124 | 7 | 104 | 59 |
|  | (Intercept) | 5.5167*** | 5.8178*** | 5.0764*** | 5.7865*** | 5.6291*** | 6.2639** | - | 5.6953*** | 6.0926*** |
| Environmental factors | Latitude | - | - | - | -0.4487** | -0.8832*** | - | - | - |  |
|  | Mean Elevation | 0.2118* | -0.2528* | - | - | - | - | - | -0.3335** | - |
|  | CV of Elevation^3^ | -0.3137** | - | - | - | - | -0.3505*** | - |  | -0.3943** |
|  | Mean Temperature | - | -0.1621 • | - | - | - | -0.6220*** | - | - | -0.5270** |
|  | Temperature Seasonality | -0.8994*** | -0.6109*** | - | - | - | -0.4243*** | - | -1.0204*** | - |
|  | Mean Precipitation | 0.6089*** | -0.2571** | 0.3205*** |  | - | 0.1265 | - | - | 0.3265*** |
|  | Precipitation Seasonality | 0.2001*** | - | -0.1861*** | -0.4733* | - | 0.2201** |  | -0.1774* |  |
| Landscape factors | CA *_Forest_* | 0.4720** | - | - | 0.5078** | - | - | - | 0.1376 | 0.2136** |
|  | CA *_Wetland_* | - | - | - | - | - | - | - | 0.5093*** |  |
|  | CA *_Built up_* | - | 0.1587 • | - | - | 0.5044* | - | - | - | - |
|  | Coh *_Forest_* | - | - | - | 0.4903* | - | - | - | - | - |
|  | Coh *_Wetland_* | - | - | - | - | - | - |  | - | - |
|  | Coh *_Built up_* | - | - | - | - | - | - | - | 0.2617*** |  |
|  | ED *_Forest_* | 0.2362** | - | 0.3588*** | - | 0.6935** | 0.1108 • | - | 0.1423 | - |
|  | ED *_Wetland_* | - | - | - | - | - | -0.2372* | - | -0.2528 • | - |
|  | ED *_Built up_* | - | -0.1411 | - | - | 0.5039* |  | - | -0.1432 | - |
|  | ES *_Forest, Built up_* | -0.3628 • | - | -0.1997 | - | - | 0.2393** | - | - | - |
|  | Large Patch Index (LPI) | - | 0.1105 | - | - | - | - | - | - | - |
|  | *R_adj_^2^* | 0.5427 | 0.5691 | 0.2380 | 0.3704 | 0.4119 | 0.3384 | - | 0.7951 | 0.4353 |

Significant differences: ^•^ P<0.1, *P<0.05, **P<0.01, and ***P<0.001 .

^1^ The sample size of the Australasian realm is not statistically representative.

^2^ Coefficient of variation of elevation.

**Table S3. The best subset selection of the Poisson regression model for amphibians in different urban regions**

|  | **Amphibians** | **World** | **Nearctic** | **Palearctic** | **Neotropical** | **Afrotropical** | **Indo-Malay** | **Australasia^1^** | **China** | **India** |
| --- | --- | --- | --- | --- | --- | --- | --- | --- | --- | --- |
|  | Number of cities | 505 | 58 | 202 | 61 | 53 | 124 | 7 | 104 | 59 |
|  | (Intercept) | 2.5500*** | 1.9111*** | 1.7714*** | 2.2439*** | 2.2739*** | 3.9102*** | - | 3.1583*** | 3.1938*** |
| Environmental factors | Latitude | - | - | - | - | -1.2444** | - | - | - | -0.6783*** |
|  | Mean Elevation | - | - | - | - | - | -0.9208** | - | -0.9863*** | - |
|  | CV of Elevation^3^ | -0.6307*** | -0.7361*** | - | - | - | - | - | -0.7752** | - |
|  | Mean Temperature | - | 1.1988*** | - | - | - | -0.9864* | - | - | - |
|  | Temperature Seasonality | -1.1108*** | - | - | - | - | -0.7715** | - | -1.1121*** | - |
|  | Mean Precipitation | 1.6582*** | 0.6332** | 1.6162*** | 2.0273*** | 1.1284* | 0.4448 • | - | - | 0.7428*** |
|  | Precipitation Seasonality | -0.3029** | -0.4525** | -0.3182* |  | - | -0.4158* |  | -0.5516*** | -0.5187*** |
| Landscape factors | CA *_Forest_* | 0.7746** | - | - | 0.8917* | - | 0.5791*** | - | 0.8282*** | 0.6069*** |
|  | CA *_Wetland_* | - | - | - | - | - |  | - | 0.3962 • | - |
|  | CA *_Built up_* | - | - | - | - | 0.8310* |  | - | - | - |
|  | Coh *_Forest_* | 0.3291* | - | - | - | 0.6324 • | 0.2789* | - | - | - |
|  | Coh *_Wetland_* | - | - | - | - | - | - | - | - | -0.0983 • |
|  | Coh *_Built up_* | - | 0.4229* | -0.5600** | - | - | - | - | 0.2949* | - |
|  | ED *_Forest_* | 0.5808*** | 0.3791* | 0.7840*** | - | 0.8422* | - | - | 0.4282** | - |
|  | ED *_Wetland_* | - | -0.3490 • | - | - | - | -1.6200*** | - | -0.3078 | - |
|  | ED *_Built up_* | - | - | 0.8187*** | - | - | - | - | - | 0.2753* |
|  | ES *_Forest, Built up_* | 1.0566** | - | ~~-~~ | 0.7531 • | - | - | - | -0.3398* | -0.5104* |
|  | Large Patch Index (LPI) | -0.4673** | - | - | - | - | - | - | - | - |
|  | *R_adj_^2^* | 0.5352 | 0.7925 | 0.5336 | 0.5003 | 0.5028 | 0.6866 | - | 0.8244 | 0.8435 |

Significant differences: ^•^ P<0.1, *P<0.05, **P<0.01, and ***P<0.001 .

^1^ The sample size of the Australasian realm is not statistically representative.

^2^ Coefficient of variation of elevation.

***Appendix C. The estimation of total sample size for best predictor selection of the Poisson regression.***

To estimate the total sample size to reach at least the same explanatory power (r^2^=0.30) as Aronson et al. (2014), we applied the power analysis (Cohen, 1988) using G*power software (Faul et al., 2007). We assumed that type I error equals 0.05 (α=0.05). The results indicated that the total sample size varies with the number of predictors selected by the *bestglm*. If *bestglm* selects all the environmental and landscape factors (number of predictors = 18), the sample size is suggested to be higher than 53 (Figure S3). On the other hand, if *bestglm* only selects one predictor, the sample size at least needs to be 13 to reach the same explanatory power (Figure S4). As such, the Australian realm, which has only 7 samples (i.e. 7 selected cities), cannot perform the best subset selection as Aronson et al. (2014).


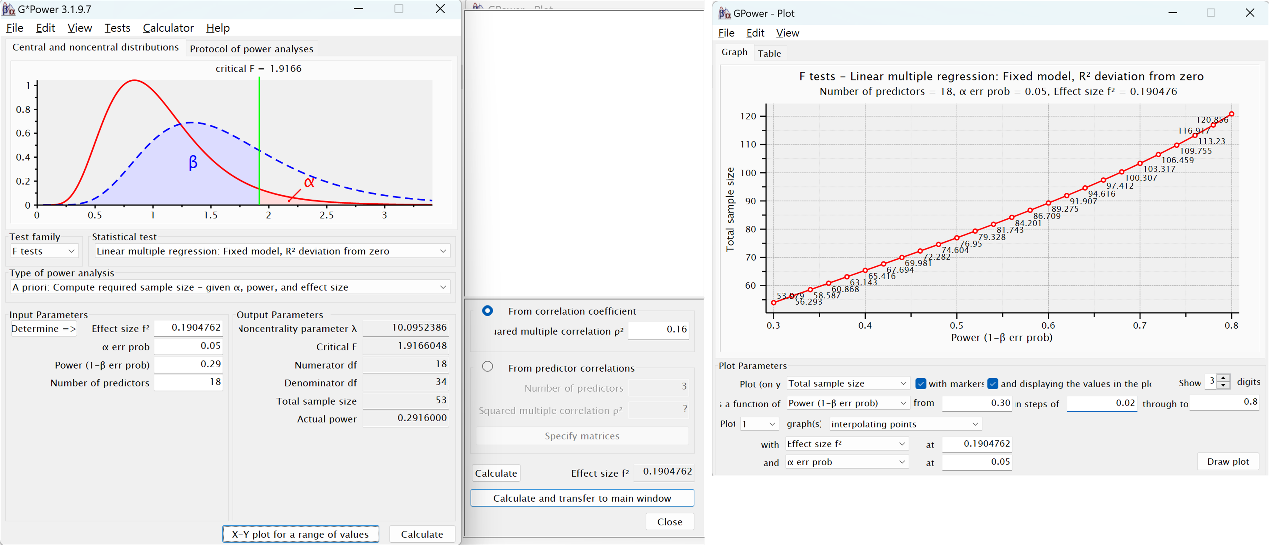


**Figure S3. Power analysis to estimate the total sample size when the number of predictors equals 18.**


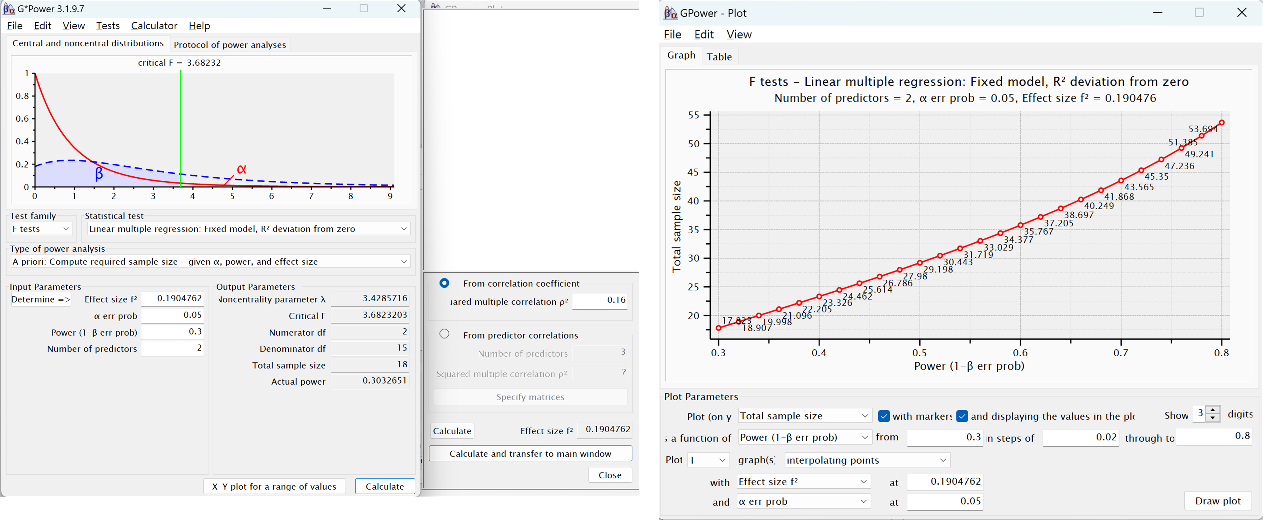


**Figure S4. Power analysis to estimate the total sample size when the number of predictors equals 1.**

References:

1. Aronson, M. F., La Sorte, F. A., Nilon, C. H., Katti, M., Goddard, M. A., Lepczyk, C. A., ... & Winter, M. (2014). A global analysis of the impacts of urbanization on bird and plant diversity reveals key anthropogenic drivers. Proceedings of the royal society B: biological sciences, 281(1780), 20133330.
2. Cohen, J. (1988). Statistical Power Analysis for the Behavioral Sciences. New Jersey: Hillsdale.
3. Faul, F., Erdfelder, E., Lang, A. G., & Buchner, A. (2007). G* Power 3: A flexible statistical power analysis program for the social, behavioral, and biomedical sciences. Behavior research methods, 39(2), 175-191.
